# Signaling by a tyrosine-sulfated peptide balances growth and osmotic stress response in rice

**DOI:** 10.64898/2026.08.04.742889

**Published:** 2026-08-05

**Authors:** Yejin Shim, Ellen Y. Rim, Jin C.-Y. Liao, Myeong-Je Cho, George Austin, Patrick W. Carlos, Sameer S. Kulkarni, Richard J. Payne, Maria Florencia Ercoli, Pamela C. Ronald

## Abstract

Peptide hormone signaling coordinates plant growth and osmotic stress responses, yet how the transition between these responses is regulated remains poorly understood. Here, we investigated the function of the rice PLANT PEPTIDES CONTAINING SULFATED TYROSINE 8 (OsPSY8) peptide in osmotic stress responses. *OsPSY8* was predominantly expressed in root tissues under non-stress conditions, with preferential expression in lateral roots where it promoted root growth. Osmotic stress rapidly reduced *OsPSY8* expression in roots through the OsWRKY24 transcription factor. Loss-of-function *ospsy8* mutants exhibited enhanced osmotic stress tolerance, whereas *OsPSY8* overexpression increased osmotic stress susceptibility. Transcriptomic analyses revealed that disruption of *OsPSY8* activated stress-responsive pathways, including those associated with lignin biosynthesis, compatible solute production, cell wall remodeling, and reactive oxygen species (ROS) scavenging, and was accompanied by increased lignin accumulation in roots. In contrast, overexpression of *OsPSY8* resulted in maintenance of growth-associated transcriptional programs while suppressing stress-responsive pathways under osmotic stress. Together, these findings identify OsPSY8 as an important regulator of the transition from growth to stress adaptation in rice and suggest that stress-induced repression of PSY signaling is required to disengage growth programs and activate adaptive responses during osmotic stress.

**Significance Statement:**

Crop survival during drought depends on the ability to transition from growth to stress adaptation. Plant peptide hormones have emerged as important regulators of this critical transition, highlighting the importance of investigating their roles and potential for improving crop resilience. We show that a rice peptide hormone regulates this transition. Under non-stress conditions, this peptide hormone, predominantly expressed in rice roots, promotes root growth while suppressing stress responses. During osmotic stress, expression of the peptide hormone decreases, resulting in activation of stress-responsive pathways, such as lignin biosynthesis and reactive oxygen species scavenging. These findings demonstrate that a peptide hormone coordinates the balance between growth and stress adaptation in rice, with broader implications for understanding and improving crop resilience.

## Full Text Availability

The license terms selected by the author(s) for this preprint version do not permit archiving in PMC. The full text is available from the preprint server.

